# Determinants of China’s health expenditure growth: based on Baumol’s cost disease theory

**DOI:** 10.1186/s12939-021-01550-y

**Published:** 2021-09-26

**Authors:** Linan Wang, Yuqian Chen

**Affiliations:** 1 School of Public Economics and Administration, Shanghai University of Finance and Economics, No. 777 Guoding Road, Yangpu District, Shanghai, 200433 China; 2 Shanghai Health Development Research Center, No. 1477 West Beijing Road, Jing’an District, Shanghai, 200041 China

**Keywords:** Health expenditure growth, Baumol’s cost disease, Determinants, Spatial Durbin model

## Abstract

**Background:**

During the past four decades, China’s total health expenditure and health expenditure per capita have both experienced a dramatic increase in growth rate. This study aims to explore the determinants of health expenditure growth and the influencing mechanism of these determinants, with considering the productivity efficiency represented by Baumol’s cost disease.

**Methods:**

Based on the longitudinal data of 30 provincial-level administrative regions in China, from 2010 to 2017, multi-variates regression models were constructed to assess the determinants, including demography, income, Baumol’s cost disease, technology, their effects on per capital total health expenditure growth and the three financing sources: government, society and out-of-pocket health expenditure. Moreover, the Spatial Durbin Model was used to analyze the influence mechanism of determinants on the increase of health expenditure across provinces.

**Results:**

Among 210 province-year growth rate observations, all of the average growth rate of total health expenditure (12.78%) was much higher than the growth rate of per capita GDP (8.06%). According to the statistical analysis, we found that:(1) Income and Baumol’s cost disease have a significant positive impact on health expenditure growth(*P* < 0.01). The impact of technical factors on government health expenditure is significantly positive. (2) The determinants affected the growth of health costs in different regions variably; the eastern region is mainly driven by Baumol’s cost disease and technical factors, while the central and western regions are mainly affected by income factors and Baumol’s cost disease. (3) There is a significant spatial spillover effect on the health expenditure growth between regions. The income factor and Baumol’s cost disease have a positive impact on the health expenditure growth in its own region as well as in other regions.

**Conclusions:**

Income and Baumol’s cost disease significantly contributed to China health expenditure growth. The health expenditure determinants showed spatial varies effect and space spillover effect on the neighborhood areas. Which indicates that a reasonable salary system should be contrasted to meet the changeling from the Baumol’s cost disease, and the necessity of equity in health resource allocation among provinces in China.

## Introduction

Since 1978, China’s total health expenditure (THE) and health expenditure per capital have both increased rapidly. The total health expenditure took around 3% of the gross domestic product (GDP) at that time and then grew to around 4% of the GDP in the 1990s. As the Chinese healthcare reform started in 2009, the percentage increased to over 5% and reached 6% in 2016. In 2018, the THE in China was 5912.19 billion Yuan ($893.43 billion), with a growth rate of 8.6%, which is higher than that of the GDP. The health expenditure per capita in 2018 was 4236.98 yuan ($640.28), with an increase of 453.14 yuan ($68.48) from 2017. The rapid growth of health expenditure posed a challenge to the sustainability of health financing as the financing level [1]. Therefore, it is necessary to understand the mechanism of health expenditure growth and provide decision-making evidence for health expenditure budgets in the new economic context.

The health expenditure growth is mainly affected by both suppliers and demanders in health services market [2–5]. Demographic factors and income factors affect health expenditure from the demand side. The change in age structure would affect the disease spectrum and further influence the needs for health services. Income level determines the ability to transform the need for health services into affordable health services, which in turn affects the health expenditure growth. Previous studies have shown that the explanatory power of income factor and demographic factor is about 50% [6, 7]. On the supply side, technology has been identified as a driver of health expenditure growth [5, 8, 9]. Besides, some researches focus on supplier productivity, that is, Baumol’s cost disease in the health sector leads to excessive health expenditures [10–14]. According to Baumol’s cost disease (BCD) theory, the entire economic industry could be simply divided into two sectors, namely “progressive sector” and “non-progressive sector” in terms of their productivity growth rates. The two sectors are quite distinguished as the latter is more labor-intensive while the former is not. In progressive sectors, the introduction of technology progress is continuously contributing to the increase of labor productivity, such as the manufacturing industry. While in the labor-intensive sector, “labor is in itself the end product” [15], which means the productivity growth is slower than the progressive sector. The health care sector is a typical “non-progressive sector” [16]. Especially high-income areas tend to have low output in the health sector, leading to a relative increase in medical costs [15, 16]. To evaluate the BCD effect, medical prices can be set as the proxy [12]. Another way is deriving Baumol variable from the perspective of unbalanced growth among different industries [13, 17].

In recent years, many studies have been conducted on the impact of Baumol’s disease on health costs, and it is found that BCD largely explains the increase in health expenditure in OECD and developed countries [11, 14, 18, 19]. Some researches have tested the effect of BCD in China, whereas the results are not consistent. Some researches focused on medical service price or wages were based on the service industry characteristics [20, 21], only a few studies using Baumol variable found that BCD also existed in the Chinese health care industry [22–24].

In this study, based on international experience and the Chinese context, we analyzed the impact of demography, income, technology, and BCD on health expenditure growth by using regional panel data. Taking the differences in health expenditure growth in various regions into account, we included a spatial variable to analyze the spatial dependence of health expenditure growth and analyzed the influence mechanisms of various factors on health expenditure growth.

## Methods

### Data source

The longitudinal data from 2010 to 2017 of 30 provinces in China mainland (Tibet was excluded due to the missing data) were collected in this study. Those data were collected from five statistics yearbooks: China Health Care Statistics Yearbook, China Population and Employment Statistics Yearbook, China Statistical Yearbook, China Demographics and Labor Statistics Yearbook and China Science and Technology Statistical Yearbook (Table 1).

**Table 1:** Different index variables meaning and the data sources

Variables			Description	Data Sources
**Dependent variables**	Health expenditure	lnTHE	Per capita actual health expenditure	China Health Care Statistics Yearbook
	Government health expenditure	lnGHE	Per capita actual government health expenditure	
	Social health expenditure	lnSHE	Per capita actual social health expenditure	
	Personal cash health expenditure	lnOOP	Per capita actual personal cash expenditure	
**Independent variables**	Demography	lnPOP65	The proportion of people aged 65 and over	China Population and Employment Statistics Yearbook
	Income	lnGDP	Real per capita GDP	China Statistical Yearbook
	Baumol variable	BV	$\varDelta log\left({C}_B(t)\right)=\frac{\left(\hat{w}-\hat{y}\right)}{l{(t)}_B}$	China Statistical Yearbook, China Demographic and Labour Statistics Yearbook
	Technology	lnRD	The ratio of R&D expenditure to regional GDP	China Science and Technology Statistical Yearbook
**Control variables**	Supplier	lnDOC	The number of health technicians per 1000 population	China Health Care Statistics Yearbook
		lnBED	The number of beds in health institutions per 1000 population	
	Demander	lnOUTP	The average number of medical visits	
		lnINP	The annual hospitalization rate	

### Health expenditure measurement

The health expenditure per capita in each province was used to represent the health expenditure level. To identify the payers, the total health expenditure (*THE)* was divided into three financing sources: government health expenditure (*GHE*), social health expenditure (*SHE*), and out-of-pocket expenditure (*OOP*). The health expenditures in the Chinese Yuan were expressed in 2010 price using GDP deflators. Following Colombier’s work (2012) [17], to avoid the seemingly not resolvable issue of determining the degree of integration of THE, we use growth rates instead of levels for the statistical analysis. For symmetry and bounding advantage, the log-difference was used to represent the growth rate instead of the period-over-period rate. For instance, the growth rate of THE was calculated by *Δ* log(*THE*_*t*_) = log(*THE*_*t*_) − log(*THE*_*t* − 1_) = log(*THE*_*t*_/*THE*_*t* − 1_). In this study, the constant e was used as the base of the logarithm in the calculation (natural logarithm).

### The health expenditure driven factors measurement

**Fig. 1:**
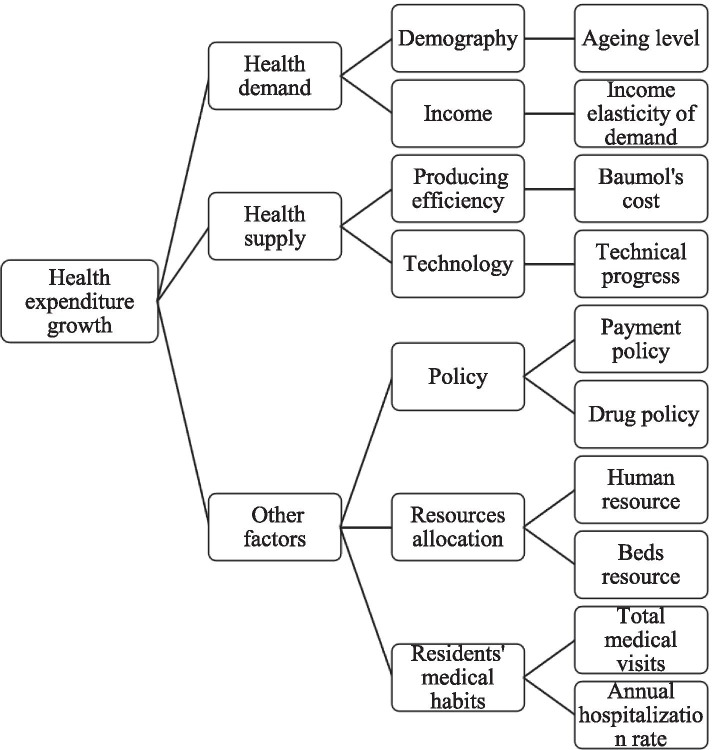
Influencing factors for health expenditure growth

According to previous studies, the driven forces of the health expenditure growth can be divided into three parts: the demand-side, the supply-side and the external context [25]. Following the framework, those determinants shown in Fig. 1 were taken into consideration in this study.

Firstly, the population structure, which mainly referred to aging and denoted as the proportion of people aged 65 and over in each province and year (*POP65*) in this study [26, 27].

Secondly, the income, which was expressed in terms of real per capita GDP based on 2010 price using GDP deflators [26–28].

Thirdly, the productivity factor, which was represented by adjusted Baumol cost in this study and was expressed as Baumol variable (BV) in the following text. The adjusted Baumol cost could be calculated by Eq. 1, according to Colombier [14, 17]:

1 $$\varDelta log\left({C}_B(t)\right)=\frac{\left(\hat{w}-\hat{y}\right)}{l{(t)}_B}$$

The left-hand side of the equation is equal to the growth rate of unit costs in the Baumol sector (refers to the health industry in this study) at time *t*. $\hat{w}$ denotes the excess of increases in wages and the productivity growth of the economy is expressed as $\hat{y}$*. l*(*t*)_*B*_ refers to the share of the Baumol sector in the total labor force at time *t*.

Fourthly, the technical factor, which was represented by the ratio of research and experimental development (R&D) expenditure in all regions to the regional GDP in this study (*RD*) [27].

### The control variables

The control variables included the number of health technicians per 1000 population, the number of beds in health institutions per 1000 population, the average number of medical visits, and the annual hospitalization rate in province *p* and time *t*, that are denoted as *DOC*_*p*, *t*_, *BED*_*p*, *t*_*, OUTP*_*p*, *t*_*, INP*_*p*, *t*_. The number of health technicians and the number of beds in medical institutions per 1000 population reflect the health resources allocation in different regions, which may associate with the rise in health expenditure, resulting from demand release or induced demand [29–31]. The average number of medical visits and the annual hospitalization rate reflect the health service utilization in different regions, which may associate with disease prevalence rate and result in health expenditure [32, 33].

### Statistical analysis

The multi-variates regression models were used to assess the effect of the determinants on the health expenditure growth. To prevent from heteroscedasticity, all variables are logarithmically transformed. The detailed info and source of each variable were shown in Table 1. To meet the precondition of stationary series, the first differences were taken for all variables.

2 $$\varDelta \ln {Y}_{p,t}=\alpha +{\beta}_1\varDelta \ln POP{65}_{p,t}+{\beta}_2\varDelta \ln {pGDP}_{p,t}+{\beta}_3 BV+{\beta}_4\varDelta \ln {RD}_{p,t}+{\eta}_p+{\tau}_p+{\varepsilon}_{p,t}$$

Where *Y*_*p,t*_ is a vector, representing a series of the health expenditure indicators, including *THE, GHE, SHE,* and *OOP*, in province *p* and year *t*. And also, to identify the regional disparity among different economic level areas, all provinces were divided into three regions: eastern, central and western. These three regions were used to represent the developed, middle-level and less developed regions in China in lots of studies [34–36]. The subscripts with other variables indicate the same province and year. The coefficient *β*_*1*_*, β*_*2*_ *β*_*3*_ and *β*_*4*_ represent the marginal effect of each determinant’s increment, including age structure, real-GDP per capita, BCD and R&D on the health expenditure. All estimates included a vector of province fixed effects (μ_p_) that control for mean differences across provinces, and year effects(**τ**_**t**_) that control for flexible year effects common to all provinces. ***ε***_***p,t***_ referred to the error term. The appropriate model type was chosen from pooled model, random effect model and fixed effect model by Breusch-Pagan Lagrange Multiplier (LM) test [37] and Hausman test [38].

Furthermore, to investigate the spillover effect across provinces, the Spatial Durbin Model (SDM) was used to analyze the influencing mechanism of each factor on the health expenditure growth across provinces.

3 $${\mathbf{Y}}_{\mathbf{p},\mathbf{t}}=\boldsymbol{\uplambda} {\sum}_{\mathbf{j}}{\mathbf{W}}_{\mathbf{p},\mathbf{j}}{\mathbf{Y}}_{\mathbf{p},\mathbf{t}}+{\mathbf{X}}_{\mathbf{p},\mathbf{t}}\boldsymbol{\upbeta} +{\sum}_{\mathbf{j}}{\mathbf{W}}_{\mathbf{p},\mathbf{j}}{\mathbf{X}}_{\mathbf{p},\mathbf{t}}\boldsymbol{\uptheta} +{\boldsymbol{\upmu}}_{\mathbf{p}}+{\boldsymbol{\uptau}}_{\mathbf{t}}+{\boldsymbol{\upvarepsilon}}_{\mathbf{p},\mathbf{t}}$$

*Y*_*p,t*_*, μ*_*p*_*, τ*_*t*_ and *ε*_*p*, *t*_ represented the same things in Eq. 2. The ***W***_*p*, *j*_ is a 30*30 spatial weighting matrix that constructed by the inverse of the distance between the capital cities of each two provinces. ***λ*** denotes the spatial autocorrelation coefficients of ***Y***_*p,t*_. ***X***_*p,t*_ is a vector, representing a series of the exogenous health expenditure determinants, including age structure, real-GDP per capita, BCD and R&D, with the associated parameters contained in the 4*1 vector ***β***. Those parameters represent the net effect of the determinants within the province *p* in year *t*. And, the parameter vector ***θ*** denotes the spillover effect of the determinants in province *p* on provinces other than province *p*. Hausman test was used to choose between the fixed effect model and random effect model.

Additionally, in a spatial Durbin model a change in a particular independent variable in a specific region has a direct effect on this region, as well as an indirect effect on the remaining regions [39]. Therefore, in this study, the total effect of those dependent variables on the health expenditure was decomposed into the direct effect, which indicates to the average health expenditure change caused by one unit change in this region’s dependent variable, and the indirect effect, which can be interpreted as the aggregate impact on the health expenditure increment of a specific region of the change in an independent variable in all other regions.

Stata MP 16.0 (Stata Corp., College Station, Texas, USA) software was used for the statistical analysis. The significant level for statistical tests was 0.05.

## Results

### Characteristics of the study provinces

The descriptive results for the provinces studied were summarized in Table 2. After calculating the growth rate with first-order lag, there were 210 province-year observations were used in our analysis. The average growth rate of real THE per capita is 12.78%, with wide regional variations (between − 0.21 and 27.94%). Among three health financing sources, the average growth rate of per capita GHE is the same as that of per capita THE, and the growth rate in different regions is between − 5.87 and 30.74%. The growth rate of per capita SHE is higher than that of THE (16.22%), and the regional growth rate is between − 19.23 and 37.51%, with the most significant difference among the three major sources. The growth rate of per capita OOP is lower than that of per capita THE and other two sources, which is only 9.46%, with regional growth ranging from − 14.45 to 29.17%.

**Table 2:** Characteristics of the 30 study provinces from 2010 to 2017

Varibales	N	Mean	SD	Min	Max
Outcome Variables					
∆lnTHE	210	0.1278	0.0510	−0.0021	0.2794
∆lnGHE	210	0.1229	0.0663	−0.0587	0.3074
∆lnSHE	210	0.1622	0.0855	−0.1923	0.3751
∆lnOOP	210	0.0946	0.0730	−0.1445	0.2917
Determinants					
∆lnGDP	210	0.0806	0.0236	−0.0238	0.1495
∆lnPOP65	210	0.0348	0.0674	−0.2512	0.2808
BV	210	0.3387	1.1036	−2.5498	7.1072
∆lnRD	210	0.0304	0.0679	−0.2559	0.2857
Control Variables					
∆lnBED	210	0.0608	0.0759	−0.4926	0.3653
∆lnDOC	210	0.0511	0.1132	−0.4837	0.5690
∆lnOUTP	210	0.0403	0.0346	−0.0454	0.1363
∆lnINP	210	0.0704	0.0469	−0.0364	0.2355

Notes: Since the prefix ∆ln denotes yearly growth rates of each variable as we introduced in Methods, there were only 7 effective observations for each province from 2010 to 2017, and 210 observations in total

The average growth rate of per capita real GDP is 8.06%, and the proportion of the population aged 65 and over, namely the degree of aging, has an average growth rate of 3.48%. The mean of BV is 0.34. That is, the difference between wage growth and output growth in the health sector is about 34%, and the average growth rate of R&D expenditure was 3.04%. In addition, the average increase in the number of beds and the number of health technicians per 1000 population was 6.08% and 5.11%, respectively, and the average increase in the number of visits per capita and hospitalization rates per 1000 population was 4.03% and 7.04%, respectively.

### The determinants’ effect on the health expenditure

As shown in columns 1 to 4 of Table 3, the impact of per capita real GDP on THE growth is significantly positive, with a coefficient between 0.799 and 0.890 (*P* < 0.01), which indicates that the income elasticity of health expenditure is less than 1. BV has a significant positive impact on THE, with coefficients between 0.245 and 0.268 (*P* < 0.01). Technology (RD) has a positive but not significant effect on THE. The impact of the proportion of the population aged 65 and over on per capita THE has changed from negative to positive when the control variables were taken into consideration. However, the proportion of elderly does not significantly associate with THE.

**Table 3:** The effects of determinants on the per capita health expenditure

	(1)	(2)	(3)	(4)	(5)	(6)	(7)
Variables	THE	THE	THE	THE	GHE	SHE	OOP
dlnGDP	0.869*** (0.194)	0.804*** (0.192)	0.799*** (0.195)	0.890*** (0.216)	1.569*** (0.319)	0.574* (0.343)	0.732** (0.326)
BV	0.0268*** (0.00383)	0.0246*** (0.00378)	0.0245*** (0.00386)	0.0260*** (0.00405)	0.0243*** (0.00571)	0.0365*** (0.00697)	0.0189*** (0.00568)
dlnRD		0.116 (0.0721)	0.115 (0.0720)	0.114 (0.0730)	0.208** (0.0927)	0.106 (0.0992)	0.0984 (0.103)
dlnPOP65			−0.0118 (0.0470)	0.00139 (0.0463)	0.0576 (0.0553)	− 0.0350 (0.0753)	− 0.0197 (0.0787)
dlnBED				−0.00597 (0.0549)	0.109* (0.0591)	−0.0840 (0.0791)	0.0546 (0.0862)
dlnDOC				−0.0297 (0.0297)	− 0.0980*** (0.0305)	0.00755 (0.0549)	−0.00400 (0.0527)
dlnOUTP				0.110 (0.126)	−0.127 (0.172)	0.122 (0.226)	0.354* (0.196)
dlnINP				−0.114 (0.0964)	−0.421*** (0.116)	0.0464 (0.164)	−0.101 (0.139)
Constant	0.0537*** (0.0162)	0.0558*** (0.0155)	0.0566*** (0.0162)	0.0541*** (0.0171)	0.0177 (0.0251)	0.105*** (0.0256)	0.0202 (0.0238)
Observations	210	210	210	210	210	210	210
R-squared	0.133	0.166	0.190	0.199	0.228	0.144	0.110

Note: Robust standard deviations in brackets, ***, ** and * indicate significant at the 1, 5 and 10% levels respectively. All variables are in first differenced logarithms and at 2000 GDP price levels; adjusted Baumol variable = (real wage rate-labour productivity) * 1/(share of Baumol sector in total employment)

As shown in columns 5 to 7 of Table 3, income has a significant positive impact on both GHE and OOP, and the income elasticity of government health expenditure (1.57, *P* < 0.01) is significantly greater than 1. While for society (0.57, *P* < 0.1) and individuals (0.73, *P* < 0.05), the income elasticity is less than 1. BCD has a significant positive impact on the three financing sources, both the GHE coefficient and OOP coefficient decreased to 0.02 (P < 0.01), and the SHE coefficient increased to 0.04(P < 0.01), comparing to the effect on THE. Meanwhile, technology showed a significant positive impact on GHE, with an elasticity coefficient of 0.21 (P < 0.05). Aging showed no significant effect on all three financing sources.

### Regional disparity of the health expenditure determinates

The influencing factors of the actual per capita THE are different in different regions as shown in Table 4. The impact of income is positive in all regions, while it is not significant in the eastern region. The income elasticity of health consumption in central and western regions is greater than 1, which means the growth of THE is significantly higher than that of GDP. BCD exists in all three regions, among which the central region is the most affected (0.04, *P* < 0.01), the western region is the second (0.03, P < 0.01), and the eastern region has a lightest effect (0.02, *P* < 0.05). The influence of technology is significantly working in the eastern region (0.29, *p* < 0.01), but not in the central and western regions, and the regional influence of demography is not significant.

**Table 4:** The effects of determinants on the per capita health expenditure in different regions

	(1)	(2)	(3)
Variables	Eastern Region	Central Region	Western Region
dlnGDP	0.372 (0.377)	1.028** (0.475)	1.201*** (0.292)
BV	0.0173** (0.00693)	0.0384*** (0.0127)	0.0268*** (0.00678)
dlnPOP65	−0.0620 (0.0642)	− 0.0128 (0.109)	0.135 (0.0847)
dlnRD	0.292*** (0.0997)	−0.0956 (0.0963)	0.0867 (0.120)
dlnBED	0.00859 (0.0592)	0.181 (0.185)	−0.0275 (0.181)
dlnDOC	−0.0285 (0.0364)	0.0103 (0.116)	−0.0643 (0.104)
dlnOUTP	0.0173 (0.160)	0.437** (0.198)	−0.162 (0.285)
dlnINP	−0.0176 (0.140)	−0.259 (0.182)	− 0.173 (0.178)
Constant	0.0854*** (0.0287)	0.0288 (0.0401)	0.0432 (0.0281)
Observations	77	56	77
R-squared	0.299	0.267	0.253
Number of prov	11	8	11

Note: Robust standard deviations in brackets, ***, ** and * indicate significant at the 1, 5 and 10% levels respectively

### The effect of spatial distribution on the determinants’ effect of the health expenditure

Table 5 provides the SDM coefficients of the explanatory factors that determine the changes in per capita health expenditures across the China, from 2001 to 2017. For per capita THE, GHE, SHE and OOP, all spatial autocorrelation coefficients ρare significant with 95% confidence intervals. That indicates the existence of the spatial autocorrelation effect for the four models. According to the Hausman test (*p* < 0.05), the fixed effects models’ results (right 2–5 columns in Table 5) were taken.

**Table 5:** Spatial Durbin Model estimation results of factors influencing health expenditure growth

	Fixed effects	Fixed effects	Fixed effects	Fixed effects	Random effects	Random effects	Random effects	Random effects
Variables	THE	GHE	SHE	OOP	THE	GHE	SHE	OOP
dlnPOP65	0.0278 (0.0536)	0.117*** (0.0334)	−0.00911 (0.0725)	0.00212 (0.0936)	0.0193 (0.0541)	0.113*** (0.0401)	−0.0538 (0.0718)	0.0052 (0.0941)
dlnGDP	0.732** (0.35)	−0.0151 (0.473)	1.237** (0.546)	0.606 (0.546)	0.533** (0.27)	0.278 (0.325)	1.151*** (0.426)	0.308 (0.465)
BV	0.0310*** (0.00518)	0.0219*** (0.00633)	0.0439*** (0.0087)	0.0254*** (0.00681)	0.0257*** (0.00388)	0.0205*** (0.00536)	0.0368*** (0.00606)	0.0215*** (0.00511)
dlnRD	0.181*** (0.0679)	0.157* (0.0924)	0.275*** (0.0743)	0.147 (0.0912)	0.151*** (0.0571)	0.253*** (0.0707)	0.155** (0.0639)	0.103 (0.0763)
dlnBED	0.00906 (0.0542)	0.176* (0.0907)	−0.203** (0.101)	0.162** (0.0699)	0.00982 (0.063)	0.154** (0.0673)	−0.141 (0.106)	0.112 (0.0971)
dlnDOC	−0.0221 (0.0273)	− 0.0886*** (0.0201)	0.0193 (0.0481)	− 0.00863 (0.0502)	− 0.0235 (0.0309)	− 0.0824*** (0.0174)	0.0353 (0.0459)	− 0.0199 (0.0601)
dlnOUTP	0.167 (0.133)	−0.232 (0.184)	0.409 (0.312)	0.484* (0.256)	−0.0272 (0.126)	−0.441*** (0.154)	0.229 (0.276)	0.139 (0.199)
dlnINP	−0.0926 (0.174)	−0.204 (0.159)	0.278 (0.299)	−0.506** (0.226)	0.00698 (0.116)	−0.0891 (0.109)	0.302 (0.233)	−0.323** (0.155)
W*dlnPOP65	0.0912 (0.426)	−0.104 (0.37)	0.908 (0.674)	− 0.0771 (0.521)	−0.384* (0.202)	− 0.467 (0.296)	−0.369 (0.323)	− 0.223 (0.359)
W*dlnGDP	5.165*** (1.503)	−2.931 (2.782)	12.33*** (3.304)	4.878 (3.553)	1.651** (0.657)	1.730* (0.996)	0.308 (0.826)	0.509 (0.932)
W*BV	0.115*** (0.0364)	0.0193 (0.0384)	0.227*** (0.0645)	0.105* (0.0583)	0.0108 (0.0125)	−0.000971 (0.0151)	0.0342 (0.0219)	−0.0125 (0.0144)
W*dlnRD	0.965* (0.576)	−0.0538 (0.597)	1.826** (0.788)	0.739 (0.626)	1.016*** (0.325)	0.403 (0.369)	1.385*** (0.348)	0.868** (0.362)
W*dlnBED	0.763 (0.483)	0.619 (0.442)	−0.412 (0.669)	1.953*** (0.672)	0.0733 (0.269)	0.0322 (0.352)	−0.0833 (0.466)	0.459 (0.432)
W*dlnDOC	−0.376* (0.215)	−0.155 (0.18)	−0.198 (0.303)	− 0.711** (0.306)	−0.145 (0.149)	− 0.0509 (0.13)	−0.313 (0.237)	− 0.106 (0.239)
W*dlnOUTP	2.026* (1.208)	1.182 (1.364)	2.225 (1.964)	4.084** (1.94)	−0.751** (0.382)	−0.082 (0.578)	−1.065* (0.646)	−0.273 (0.643)
W*dlnINP	−1.654 (1.111)	−1.444* (0.758)	−1.058 (1.861)	−2.985** (1.383)	−0.341 (0.235)	−0.468 (0.315)	−0.469 (0.372)	0.0966 (0.454)
rho	−0.434** (0.205)	−0.666** (0.261)	−0.890*** (0.225)	− 0.467*** (0.175)	−0.087 (0.178)	0.412*** (0.134)	−0.377* (0.197)	0.14 (0.106)
lgt_theta					14.84*** (0.217)	14.73*** (0.372)	17.67*** (0.257)	16.26*** (0.32)
sigma2_e	0.00151*** (0.00019)	0.00168*** (0.000302)	0.00467*** (0.000595)	0.00357*** (0.000546)	0.00177*** (0.000228)	0.00212*** (0.000374)	0.00561*** (0.000714)	0.00441*** (0.000601)
Constant					−0.00657 (0.0283)	−0.0434 (0.0485)	0.137*** (0.0447)	−0.0152 (0.0405)
Observations	210	210	210	210	210	210	210	210
R-squared	0.193	0.007	0.032	0.087	0.316	0.512	0.217	0.165
Number of prov	30	30	30	30	30	30	30	30

Note: Robust standard deviations in brackets, ***, ** and * indicate significant at the 1, 5 and 10% levels respectively

Generally, income positively affect the per capita THE with the elasticity coefficients of 0.73, that are significant with the 95% confidence intervals. BCD and technology have a positive impact on per capita THE, significant at the 95% confidence intervals. However, the demographic factors have no significant on the per capita THE.

Furthermore, according to the SDM results, SHE has significantly spatial spillover effects. That can be proved by the elasticity coefficient of the spatial lag terms of per capita GDP (12.33, *p* < 0.01), BV (0.23, p < 0.01) and the technology variables (1.83, *p* < 0.05).

Table 6 shows the effect decomposition of factors influencing THE, GHE, SHE and OOP, based on the SDM, according to those fixed effect models in Table 5. Income and BCD have significant positive impact on the growth of THE, including direct effects, indirect effects and total effects, that is, income and productivity have strong spatial spillover effects. The direct effect of technology on THE growth is significant (0.16, *P* < 0.05), but the indirect effect is not significant. The effect of aging on THE growth is not significant, which is consistent with results above.

**Table 6:** Decomposition of spatial effect of the determinants on the health expenditure

	THE			GHE			SHE			OOP		
	(1)	(2)	(3)	(4)	(5)	(6)	(7)	(8)	(9)	(10)	(11)	(12)
Variables	Direct effects	Indirect effects	Total effects	Direct effects	Indirect effects	Total effects	Direct effects	Indirect effects	Total effects	Direct effects	Indirect effects	Total effects
dlnPOP65	0.0285 (0.0541)	0.103 (0.311)	0.131 (0.321)	0.123*** (0.0375)	−0.0847 (0.243)	0.0380 (0.234)	− 0.0377 (0.0700)	0.548 (0.380)	0.511 (0.390)	0.00694 (0.0935)	−0.0171 (0.365)	− 0.0101 (0.402)
dlnGDP	0.629* (0.349)	3.699*** (1.336)	4.327*** (1.300)	0.0444 (0.478)	−1.742 (1.742)	−1.698 (1.609)	0.834 (0.561)	6.508*** (1.906)	7.342*** (1.878)	0.496 (0.560)	3.492 (2.732)	3.988 (2.529)
BV	0.0296*** (0.00460)	0.0783*** (0.0299)	0.108*** (0.0314)	0.0223*** (0.00587)	0.00533 (0.0238)	0.0276 (0.0254)	0.0379*** (0.00797)	0.110*** (0.0399)	0.148*** (0.0413)	0.0241*** (0.00634)	0.0705* (0.0422)	0.0946** (0.0417)
dlnRD	0.164** (0.0653)	0.671 (0.414)	0.835* (0.446)	0.159* (0.0934)	−0.0899 (0.384)	0.0687 (0.370)	0.223*** (0.0600)	0.920** (0.409)	1.143*** (0.423)	0.133 (0.0844)	0.479 (0.439)	0.611 (0.477)
dlnBED	−0.00317 (0.0568)	0.548 (0.369)	0.545 (0.356)	0.163* (0.0890)	0.308 (0.249)	0.471* (0.281)	−0.191* (0.111)	−0.149 (0.398)	− 0.340 (0.353)	0.130* (0.0738)	1.334** (0.547)	1.464*** (0.524)
dlnDOC	−0.0150 (0.0297)	−0.263 (0.173)	−0.278* (0.157)	− 0.0855*** (0.0196)	−0.0539 (0.109)	− 0.139 (0.112)	0.0273 (0.0548)	− 0.117 (0.195)	−0.0899 (0.167)	0.00544 (0.0523)	−0.496** (0.240)	− 0.491** (0.217)
dlnOUTP	0.135 (0.139)	1.488* (0.904)	1.623* (0.949)	−0.262 (0.183)	0.858 (0.839)	0.595 (0.898)	0.341 (0.312)	1.142 (1.117)	1.483 (1.201)	0.406 (0.260)	2.808** (1.422)	3.215** (1.586)
dlnINP	−0.0698 (0.189)	−1.186 (0.877)	−1.256 (0.810)	−0.183 (0.150)	− 0.772* (0.448)	−0.955** (0.475)	0.309 (0.338)	−0.702 (1.150)	− 0.393 (0.990)	−0.460* (0.252)	−1.987* (1.179)	−2.448** (1.054)

Note: Columns 1–3 show growth in real health expenditure per capita, columns 4–6 show growth in real government health expenditure per capita, columns 7–9 show growth in real social health expenditure per capita, and columns 10–12 show growth in personal cash health expenditure per capita. Robust standard deviations are in parentheses, with ***, ** and * indicating significant at the 1, 5 and 10% levels respectively

Aging only has a significant direct effect on GHE growth (0.12, *P* < 0.01), while income has a significant positive spatial spillover effect on SHE growth (6.51, P < 0.01). BCD has a significant direct effect on GHE growth in its own region (0.2, P < 0.01), which indicates that there is no spatial spillover effect. And the direct and indirect effects on SHE and OOP are both significantly positive, and there is a positive spatial spillover effect. The direct effect and indirect effect of technology on SHE are both significantly positive, which means that there is a spatial spillover effect, and its’ direct and indirect effects on OOP growth are not significant.

## Discussion

### Income and Baumol’s cost disease drive China health expenditure growth, while aging and technology effects are insignificant

Income was the most important factor affecting health expenditure growth. Consistent with developed national level and OECD panel studies [40–42], the income elasticity was less than 1 in China, which means health care is a kind of necessity, as well as for society and individuals. Notably, the elasticity coefficient of GHE was greater than 1, which reflected the trend of increasing government investment in health.

BCD was significant in China’s health industry, which is consistent with OECD and other developing country studies using health accounting data [10, 11, 14, 43, 44] or calculated health expenditure data [23, 24]. The particularity of the health industry determines its relatively slow improvement on production efficiency, while the rigid wage demand increases the health institutions’ cost, leading to the increase in health expenses. BCD is mainly due to relatively higher wage growth than output growth, reflecting the relatively low output efficiency in the health sector. On the one hand, we should pay attention to the salary system in the health industry, establish a salary system in line with the characteristics of health industry and reasonably determine hospital salary level based on the current level. On the other hand, we can focus on the internal cost control in the medical service system, such as strengthening cost accounting, improving economic management level, and controlling medical expenses growth through performance appraisal.

Aging effect on health expenditure growth was not significant, which could be attributed to the following two reasons: one was data sources and standards. Previous studies on the impact factors of health expenditure in China mostly used the national level health expenditure accounting data or single provincial data, and study results showed that aging had a positive impact on health expenditure [45, 46]. Instead, in this study, the provincial-level health expenditure accounting data was used, and the insignificant effect of aging was consistent with pervious studies conducting with the same statistical standards [47], but different from others with shorter health accounting panel data [48, 49]. The second was the model setting mode. Since all variables in this study were incremental, with the deepening of aging degree, the growth trend of per capita real health expenditure did not show an apparent increase.

Technology had an overall insignificant impact on health expenditure, but its impact on government health expenditure was significantly positive. With the in-depth advancement of medical reform in China, local governments have increased their investment in health care in recent years. The investment in the medical service system mainly focuses on facilities and equipment, that investment was largely associated with the medical technology progress.

### The influencing factors of health expenditure in different regions are different

Health expenditure growth in the eastern region was mainly driven by BCD and technological progress, while health expenditure growth in the central and western regions was due to local economic development and BCD. Among all the determinants, BCD shows different effect on health expenditure in different regions. In consistent with Ho but different from Yuan [22, 24], the impact of BCD was greatest in the central region, followed by the western region, and the eastern region had the least impact. That may due to the greater labor mobility in eastern China, which slowed the wage growth in the service sector in that region and led a relatively small impact from BCD. In eastern and central regions, BCD had a significant positive effect on three major health expenditures growth, while in western regions, BCD only has a positive effect on OOP expenditure growth.

### Health expenditure growth has a significant space spillover effect

It is found that spatial interaction or spatial dependence is present in health expenditure across regions in China, which is consistent with US state-level studies [50], and some studies on government health expenditure, health insurance financing or OOP spillover effect in China [51–54]. In combination with the spatial effect, various factors have different influence mechanisms on health expenditure growth. Income has shown a significant stimulus to the growth of health expenses in the local and other regions. BCD also had a significant spatial spillover effect. As health sector output efficiency lags behind wage growth, resulting in higher health costs affecting health costs growth in the region as well as elsewhere. Technological progress had only a positive impact on local health costs growth, indicating that technology has a promoting effect in the local area, but not in other regions.

The respective influencing factors of three major health expenditures showed that the spillover effect of BCD was mainly reflected in social health expenditures and OOP health expenditures. On the one hand, the production efficiency in the health industry directly affects the equity and affordability of medical expenses. On the other hand, it affects the sustainability of medical insurance funds in neighboring areas. Income and technology had significant spillover effects on social health expenditure growth, which suggests that income level and technological development in different regions should be fully considered in medical insurance financing design.

The spatial dependence of health expenditure reflects the uneven distribution of medical resources. On the one hand, medical services fairness can be improved by formulating unified medical quality control standards and strengthening exchanges and cooperation among medical institutions, so as to coordinate high-quality medical resources distribution in large cities and strengthen advantageous resources output in medical centers. On the other hand, local governments should improve China’s hierarchical medical system to keep common and frequently occurring diseases in primary medical institutions, which will reduce the burden on high-level hospitals.

## Conclusions

According to the statistical analysis with the latest province-level health accounting data in China, from 2010 to 2017, we found that: (1) income and BCD had a significant positive effect on health expenditure growth. (2) Health expenditure in the eastern region were mainly driven by BCD and technology, while those in the central and western regions were mainly affected by income and BCD. (3) There was a significant space spillover effect on health expenditure growth, income and BCD had a significant incentive to health expenditure growth locally and elsewhere. We can control the unreasonable health expenses growth by constructing a reasonable salary system, strengthening internal cost control, perfecting the hierarchical medical system, and improving medical resources fairness. It is hoped to provide references for other countries to control the excessive growth of health costs.

## Data Availability

The datasets used during the current study are available from the corresponding author on reasonable request.

## Abbreviations

BCD: Baumol’s cost disease
THE: total health expenditure
GHE: government health expenditure
SHE: social health expenditure
OOP: out-of-pocket health expenditure
BV: Baumol variable
R&D: research and experimental development\
SDM: Spatial Durbin Model.
